# EGFL7 meets miRNA-126: an angiogenesis alliance

**DOI:** 10.1186/2040-2384-2-9

**Published:** 2010-06-08

**Authors:** Iva Nikolic, Karl-Heinz Plate, Mirko HH Schmidt

**Affiliations:** 1 Institute of Neurology (Edinger Institute), Johann Wolfgang Goethe University School of Medicine, Heinrich-Hoffmann-Str. 7, Frankfurt am Main, D-60528, Germany

## Abstract

Blood vessels form *de novo *through the tightly regulated programs of vasculogenesis and angiogenesis. Both processes are distinct but one of the steps they share is the formation of a central lumen, when groups of cells organized as vascular cords undergo complex changes to achieve a tube-like morphology. Recently, a protein termed epidermal growth factor-like domain 7 (EGFL7) was described as a novel endothelial cell-derived factor involved in the regulation of the spatial arrangement of cells during vascular tube assembly. With its impact on tubulogenesis and vessel shape EGFL7 joined the large family of molecules governing blood vessel formation. Only recently, the molecular mechanisms underlying EGFL7's effects have been started to be elucidated and shaping of the extracellular matrix (ECM) as well as Notch signaling might very well play a role in mediating its biological effects. Further, findings in knock-out animal models suggest miR-126, a miRNA located within the *egfl7 *gene, has a major role in vessel development by promoting VEGF signaling, angiogenesis and vascular integrity. This review summarizes our current knowledge on EGFL7 and miR-126 and we will discuss the implications of both bioactive molecules for the formation of blood vessels.

## Introduction

Vasculogenesis and angiogenesis are basic processes through which new blood vessels arise. Vasculogenesis entails the differentiation of mesodermal cells into endothelial precursor cells (angioblasts). This is followed by the formation of primitive blood vessels which are subsequently refined and transformed into a functional vascular network by the process of angiogenesis [1,2]. The execution of these tightly regulated programs depends on a vast array of factors whose identification has been a prime focus of cardiovascular research in the last two decades. One of the newly described molecular players in the field of blood vessel formation is EGFL7, a protein also known as VE-statin, MEGF7, Notch4-like protein or Zneu1. Initial studies on the role of EGFL7 in the vascular system were compiled by Soncin et al., who showed that EGFL7 inhibited the migration but not the proliferation of human aortic smooth muscle cells *in vitro *[3]. This indicated that EGFL7 might have a role in vessel maturation. However, a keynote publication of Parker et al. in 2004 established the role of *egfl7 *as an important tubulogenic factor in the process of vasculogenesis [4].

### EGFL7 structure, temporal and spatial distribution

**Figure 1:**
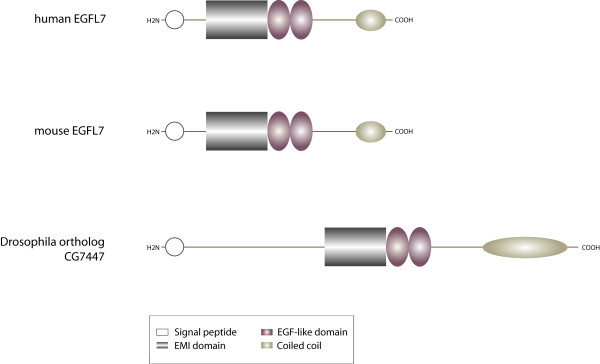
**Domain organization of EGFL7**. The modular assembly of EGFL7's domains is conserved among vertebrate species. The protein contains an N-terminal signal secretion peptide, an EMI domain, two EGF-like domains (the distal one binds Ca^2+^) and a C-terminal coiled-coil structure. The EGFL7 orthologue CG7447 in *Drosophila melanogaster *shares the same overall structure but differs in total length.

**Figure 2:**
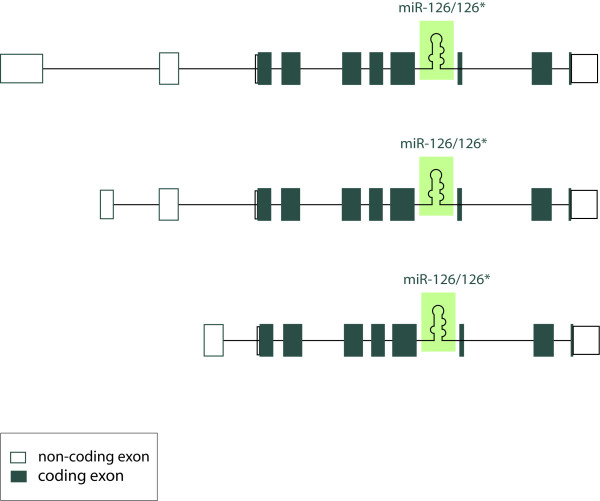
Localization of miR-126/miR-126* within the primary human EGFL7 transcript. This is a schematic representation of three alternative primary human EGFL7 transcripts, which initiate from separate promoters but contain the same open reading frame. Non-coding exons are indicated in uncolored boxes, coding exons in colored ones. miR-126 and miR-126* are localized within intron 7 of EGFL7 in all vertebrates.

Initially, EGFL7 was described as a 30 kD protein exclusively expressed by vascular endothelial cells [3]. The protein is conserved among vertebrates but an orthologue is also found in *Drosophila melanogaster *(CG7447) (Figure 1). Two alternative splice variants of EGFL7 have been described in the mouse genome [3]. The transcripts contain the same protein coding region and display 73% sequence identity to human EGFL7 on the protein level [5]. The analysis of human *egfl7 *revealed the existence of three alternative isoforms that contain the same open reading frame but are transcribed from separate promoters [6] (Figure 2).

In vertebrates the *egfl7 *gene encodes the biologically active miRNAs miR-126 and miR-126*. Both are relevant for the development of the cardiovascular system and have been implicated in cardiovascular diseases as well as the formation of cancer [7-10]. The structural assembly of the encoded protein is modular with an N-terminal signal secretion peptide, followed by an Emilin-like domain (EMI) that is succeeded by two EGF-like domains. The distal domain belongs to the Ca^2+^-binding EGF-like domains [3,4]. Typically these domains are associated with multimeric proteins involved in protein-protein interactions [11] and have been described in secreted proteins that partially incorporate into the ECM [11,12].

Initial expression analyses indicated EGFL7 is restricted to the vascular endothelium at all stages of mouse development [5,13]. However, later work by Campagnolo et al. demonstrated the presence of EGFL7 in the primordial germ cells during homing into the gonads [14]. Most importantly, EGFL7 is expressed within the neurons of adult mice, which suggests EGFL7 serves diverse biological functions in various tissues and not only in the vascular system [15]. EGFL7 becomes detectable at the blastocyst stage during mouse development with a marked increase in expression levels at embryonic day E7.5 to E8.5. Subsequently, expression remains at a constant level [3,5]. Upon birth, EGFL7 becomes downregulated in the vascular system and significant levels of the protein are only maintained in a subset of vessels in the lung, heart, kidney, spleen and uterus. High expression levels of EGFL7 are regained upon the onset of physiological angiogenesis, e.g. in the uterus during pregnancy [13] or alternatively, under pathological conditions of vessel formation subsequent to arterial injury [13], hypoxic insult [16] or in human solid tumors [4]. In sum, the striking temporal and spacial expression pattern of EGFL7 indicates a function of this protein in blood vessel formation and remodeling.

### EGFL7 in the extracellular matrix

The ECM is one of the key components of the vascular system as it maintains the organization and regulation of endothelial cells [17]. The ECM supports endothelial cell proliferation, migration, survival and morphogenesis during blood vessel formation. Primarily, this occurs through adhesive interactions with integrins on the endothelial cell surface. In addition, ECM proteins may function to sequester angiogenic cytokines allowing for the coordination of signals transduced via growth factor receptors and integrins [18,19]. Several parameters indicate EGFL7 is associated with the ECM. First, when overexpressed in fibroblasts, EGFL7 is mainly detected in the ECM fraction and the cell lysates and only little amounts of the protein are spotted in the conditioned medium. This suggests EGFL7 remains largely attached to the cell surface, most likely through interactions with ECM molecules [20]. Furthermore, the deposition of EGFL7 in the ECM is facilitated by certain types of matrix proteins, such as fibronectin and collagen type I, whereas laminin and collagen type IV do not exert such an effect [20]. Last, EGFL7 has been shown to promote endothelial cell adhesion and focal complex formation although not as efficiently as classical ECM molecules like collagen or fibronectin do [4]. In a recent study, Lelievre et al. used a transgenic mouse model to express EGFL7 in the epidermis and detected a colocalization of EGFL7 and elastic fibers in the ECM of epidermal blood vessels. Such they identified EGFL7 as a negative regulator of vascular elastogenesis [21]. Elastic fibers are the largest structures in the ECM with a very complex organization. Elastin as one of the key fiber components is produced in a process that includes cross-linking of tropoelastin molecules by a family of lysyl oxidases (LOXs) [22]. The colocalization of EGFL7 with the elastic fibers caused an inhibition of the enzymatic activity of LOXL2 by direct interaction and interfered with the process of elastin deposition [21]. Enzymes of the LOX family cross-link elastin and collagen [23] therefore one may speculate that EGFL7 participates in the shaping of the ECM thereby indirectly affecting endothelial cell functions such as migration. Particularly, these findings are interesting because EGFL7 molecules harbor conserved domains that are typically associated with ECM proteins. The EMI domain is a cysteine-rich repetitive element often detected in extracellular proteins that form multimers, e.g. emilin-1 and emilin-2. Likewise, EGF-like domains are frequently found in extracellular proteins like the constituents of elastic fibers [11,24] or common ECM molecules like laminin and tenascin [12]. Taken together, data suggest EGFL7 as a putative novel component of the ECM giving clues as to how it affects endothelial cells.

### EGFL7 in vasculogenesis

**Figure 3:**
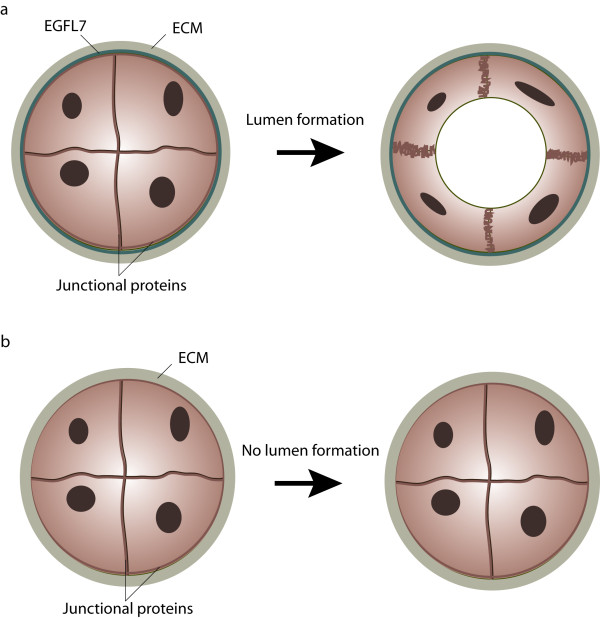
**Role of EGFL7 in lumen formation**. (**a**) During primitive plexus formation angioblasts assemble into vascular cords which are subsequently transformed into vascular tubes. Cord-to-tube transition is characterized by cell polarization, redistribution of junctional proteins and eventually, changes in cell shape. EGFL7 is secreted by endothelial cells into the extracellular matrix, where it affects the process of lumen formation. (**b**) In the absence of EGFL7 angioblasts fail to separate, which leads to the formation of vessels lacking a vascular lumen.

Early in embryonic development several inductive cues, e.g. members of the fibroblast growth factor (FGF) or the bone morphogenetic protein (BMP) families, initiate the differentiation of hemangioblasts from the undifferentiated mesoderm [25,26]. Hemangioblasts form aggregates in which the inner cells develop into hematopoietic precursors and the outer population eventually gives rise to endothelial cells. Subsequently, endothelial precursor cells or angioblasts differentiate and assemble into a primitive vascular network. Commonly, this process is referred to as vasculogenesis [27]. Solid evidence for a role of *egfl7 *in the vasculature was presented by Parker et al. in their study concerning the role of *egfl7 *in vascular tube formation during vasculogenesis [4]. During the process of primitive plexus formation cells do not directly assemble into tubes but first aggregate side by side to form cord-like structures which in later stages acquire a vascular lumen through the process of tubulogenesis [28]. Gradually, angioblasts start to separate during cord-to-tube transition due to the relocalization and modification of cell junctions, which is then followed by the formation and increase of extracellular space between adjacent cells. Eventually, cells undergo extensive morphological changes to finally shape the tubes [4,29]. This defined sequence of events was impaired in zebrafish embryos upon deletion of EGFL7 (Figure 3). More specifically, angioblasts did not segregate and retained tight junctions across the space where the vascular lumen was supposed to form. This impairment resulted in the absence of lumens in the majority of vessels [4]. This particular phenotype ascribed an important role to EGFL7 in the process of tubulogenesis but the underlying molecular mechanism remained unclear. The authors showed EGFL7 promoted adhesion of human umbilical vein endothelial cells, however, not as efficient as conventional ECM molecules. In light of the finding that several cell types favor cell migration over strict adhesion under intermediate cell adhesion conditions [30], one might speculate that EGFL7 creates a microenvironment that facilitates the local motility of endothelial cells during tube formation. Nevertheless, data involving the effect of EGFL7 on endothelial cell migration remain controversial and ambiguous. It has been reported that EGFL7 promoted the migration of several mouse endothelial cell lines *in vitro *[13], while other studies showed that EGFL7, alone or in combination with other ECM proteins, did not affect the migration of endothelial cells in a modified Boyden chamber nor random HUVEC motility monitored by live cell microscopy [3,4,20]. A recent study by Schmidt et al. postulated EGFL7 stimulates collective migration of endothelial cells within an angiogenic sprout by maintaining the correct spatial distribution of the cohort [20]. In addition to the investigation of the role of EGFL7 in endothelial cell motility and migration, it might be interesting to study the arrangement of tight junctions in EGFL7^-/- ^zebrafish embryos because it has been shown that cell-matrix interactions control the distribution of cell-cell adhesion molecules and polarity proteins during lumen formation [31]. Indeed, data recently published by Durrans et al. demonstrated that the deletion of EGFL7 in an embryoid body model resulted in the formation of abnormal endothelial cell aggregations designated as CD31+ sheets, which lacked a full basement membrane and cell junctions [32].

### EGFL7 in angiogenesis

In contrast to vasculogenesis, the process of angiogenesis involves the formation of new blood vessels from the pre-existing vascular network [33]. In most experiments, angiogenic sprouting is studied although angiogenesis also proceeds through intussusceptions [34,35]. Angiogenic sprouting involves a number of tightly regulated steps: vasodilatation and endothelial permeability, endothelial cell proliferation and migration, lumen formation as well as endothelial cell survival and vessel maintenance [36]. Due to the role of EGFL7 in tubulogenesis [4] it is reasonable to assume a comparable role for EGFL7 in angiogenic sprouting. In order to investigate this matter, Schmidt et al. created two independent mouse lines harboring EGFL7 deletions [20]. The first line was generated by application of a retroviral gene trap vector which was inserted in intron 2, upstream of the translation initiation codon in exon 3, whereas the second mouse line was created by homologous recombination resulting in the removal of the DNA region lasting from exon 5 to exon 7. 50% of the corresponding EGFL7^-/- ^embryos died *in utero *and the surviving mice displayed severe vascular developmental defects in most tissues analyzed. More specifically, a delay in blood vessel expansion within coronary, retinal and cranial vascular beds was observed in the absence of EGFL7. Surprisingly, this phenotype resolved in later developmental stages and a functional vasculature formed in all of the examined organs. This interesting observation may be explained by the upregulation of EGFL8, also known as VE-statin-2 or NG3, which is an EGFL7 homolog. Both proteins share the same overall domain structure and a similar expression pattern with an overall protein sequence homology of 35%. The highest levels of EGFL8 are detected in kidney, brain, thymus and lung [5]. The fact that *egfl8 *is present in mouse but not in zebrafish could potentially account for the striking and persistent phenotype observed in zebrafish embryos upon EGFL7 depletion that has not been detected in EGFL7^-/- ^mice [4].

In order to unravel the cellular basis for the observed defects in murine vessel growth, Schmidt et al. performed a detailed analysis of angiogenic sprout morphology [20]. Namely, they studied angiogenic sprouting, which proceeds through the coordinated actions of two cell types: tip cells and stalk cells. Tip cells sense a VEGF gradient in the surrounding environment and extend filopodia, which leads the angiogenic sprout in a specific direction. The trailing stalk cells on the other hand proliferate and support sprout elongation [37]. Eventually, endothelial cells reacquire a quiescent phenotype, recently referred to as phalanx cells, which mediate the stabilization of newly formed vessel [38].

In wild type animals tip and stalk cells organize within a single cell layer, while in EGFL7 knock-out mice both cell types form multiple cell layers similar to the cell aggregates observed in EGFL7 knock-down zebrafish embryos [4]. Immunohistochemical staining of collagen IV revealed that this ECM molecule, which is typically localized in the basal membrane, was found within these enlarged sprouts and was detected between the adjacent endothelial cells. This suggests that endothelial cells lacking EGFL7 failed to properly detect the sprout boundaries. Previously, it has been shown that EGFL7 supports the weak adhesion of endothelial cells [4], suggesting EGFL7 creates an environment where cells easily attach and detach until properly positioned. In the absence of EGFL7, however, cells may clump together and build oversized sprouts resulting in an impaired migration and delayed vascularization as observed in EGFL7^-/- ^mice.

**Figure 4:**
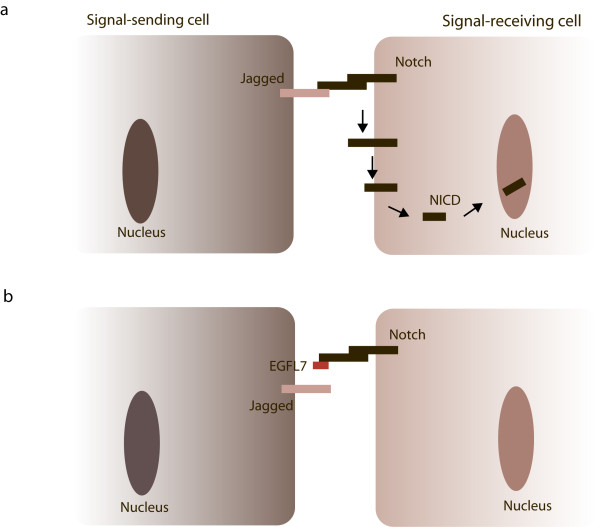
**EGFL7 and Notch signaling**. Notch receptors are expressed on the cellular surface as heterodimers connected by non-covalent interactions. (a) Upon binding of Jagged-type ligands expressed on neighboring cells *in trans*, Notch receptors undergo a series of proteolytic cleavage events releasing the NICDs of the receptors and allowing their translocation into the nucleus. (b) If EGFL7 is present in the surrounding of the cells, it competes with Jagged for Notch binding thereby attenuating Notch signaling.

A recent study of Schmidt et al. provided another compelling clue for resolving the function of EGFL7 protein in angiogenesis [15]. Most interestingly, a link between EGFL7 and Notch signaling has been unraveled. The Notch pathway is evolutionarily conserved and governs fundamental processes such as development, cell-fate determination and differentiation [39]. Notch-mediated signal transduction is based on several key molecules: four Notch receptor isoforms (Notch1-4) and five canonical ligands of the Delta (Dll1, 3 and 4) or the Jagged type (Jagged1 and 2) [40]. The role of Dll3 has been controversially discussed and it is not considered a *bona fide *Notch ligand [41]. Signaling in this pathway proceeds through ligand-receptor binding which leads to intra-membrane proteolysis of the Notch receptor and release of the Notch intracellular domain (NICD). The activated C terminus of the Notch receptor translocates into the nucleus, where it associates with the DNA-binding protein CSL to promote transcription [42]. Interestingly, Schmidt et al. demonstrated the binding of EGFL7 to all four Notch receptors and suggested that EGFL7 acts as a modulator of Notch receptor activation by its canonical ligands Jagged1 and Jagged2 [43]. This might have important implications in the vascular system since the Notch pathway plays an indispensable role in the tip-stalk cell decision during angiogenic sprouting [44-47]. Namely, high levels of Dll4 expressed on filopodia-rich tip cells that lead the angiogenic sprout (see EGFL7 in angiogenesis) activate Notch receptors present on the adjacent stalk cells. Although it is not completely understood how this suppresses the tip cell phenotype, it is believed that the activation of the Notch pathway causes a downregulation of the expression of VEGF receptors and consequently dampens the response of stalk cells to the surrounding VEGF [45,47]. Recently, Jagged1 has been suggested to be exclusively expressed on stalk cells where it antagonized Dll4-mediated activation of Notch receptors. In turn, elevated levels of Jagged1 led to increased angiogenesis and tip cell numbers [48]. Likely, members of the family of Fringe glycosyltransferases mediated the differential activation of Notch receptors by the enhancement of Notch activation in response to Delta-like ligands, which was paralleled by a reduction of Notch activity in response to Jagged-type ligands [49]. Interestingly, a similar pattern of differential Notch pathway modulation was reported by Schmidt et al. They demonstrated that EGFL7 acted as an inhibitor of Notch receptor activation by competition with Jagged-type ligands (Figure 4) and presented preliminary data suggesting a selective binding of EGFL7 to Dll4. These findings offered the possibility that EGFL7 affects vascular Notch signaling during angiogenic sprouting.

### miR-126 in angiogenesis

Recently, findings on the role of EGFL7 in angiogenesis have been supplemented by several papers describing a role of miR-126 in the vascular system [6,50,51]. Collectively, miRNAs represent a class of about 22 nucleotide long, non-coding RNAs that have been recognized in recent years as important regulators of gene expression [52]. Predominantly, miRNAs repress protein expression by the inhibition of protein translation and to a lesser extent by mRNA degradation [53]. Mounting evidence indicates the importance of miRNAs in blood vessel formation by the regulation of endothelial and smooth muscle cell functions [54,55]. Most interestingly, miR-126 is located in intron 7 of *egfl7 *(Figure 2). Currently, it is the only miRNA known to be specifically expressed in the endothelial cell lineage and hematopoietic progenitor cells [6,50,56,57]. A significant role of miR-126 for tumor development has been suggested by various studies describing a downregulation of miR-126/miR-126* in primary tumors and cancer cell lines [10,58-60]. The restoration of miRNA-activity by overexpression of miR-126/miR-126* led to a reduction of overall tumor growth, migration and invasiveness [10,58,59], in part by inhibition of cancer cell proliferation.

Two papers were published back-to-back studying the function of miR-126 in vessel development by loss of function experiments either in zebrafish [6] or mouse [50]. In both cases loss of miR-126 caused similar phenotypes *in vivo*: impaired endothelial cell migration during vessel growth as well as collapsed vessel lumens and a compromised endothelial tube organization. Analyses on the molecular level revealed that miR-126 repressed SPRED1 and PIK3R2, which negatively regulate VEGF signaling via the MAP kinase and PI3 kinase pathways. Previous EGFL7 deletion strategies affected the region encoding miR-126 and most likely, affected the transcription of miR-126 as well. Although miR-126 levels have not been measured in the initial EGFL7 knock-out study, concerns were raised on the data describing the effects of EGFL7 on angiogenic sprouting [20]. Indeed, vascular defects seen in the miR-126^-/- ^model were very reminiscent of the original EGFL7 knock-out analyses. To further address this problematic issue, Kuhnert et al. generated two mouse lines, one with an EGFL7 protein deletion and one with a miR-126 deletion that did not show any cross-perturbation of EGFL7 and miR-126 [51]. The miR-126^-/- ^mice were born at a frequency of 50% embryonic lethality and displayed a range of vascular defects similar to the ones described by Schmidt et al. in EGFL7^-/- ^mice [20]: hemorrhage, delayed postnatal retinal and cranial angiogenesis and the presence of abnormally thickened endothelial sprouts. Surprisingly, Kuhnert's EGFL7^-/- ^mice were phenotypically normal and born at the expected frequency. Further, these mice did not display any of the vascular defects seen in the previously published miR-126^-/- ^mice. Taken together, this strongly supports the possibility that the phenotype of EGFL7^-/- ^mice described by Schmidt et al. actually reflected the loss of function of miR-126. Yet another EGFL7 loss-of-function study putatively affecting miR-126 expression was presented by Parker et al. who described EGFL7 as an important factor for vascular tube formation during zebrafish development [4]. Indeed, the EGFL7 knock-down phenotype observed in zebrafish embryos in this study appears to resemble the one detected upon miR-126 knock-down [6]: hemorrhage as well as the presence of vessels with compromised lumens. However, both phenotypes display distinct differences, e.g. the block in initial lumen formation due to the failure of angioblast separation in EGFL7 knock-down embryos. In miR-126 knock-down embryos lumens are formed but collapse at later stages of zebrafish development. Most unfortunately, endogenous levels of miR-126 have not been assessed in the EGFL7 knock-down study but a downregulation of miR-126 seems less likely in this setting. First, two morpholinos have been used to knock-down EGFL7 in zebrafish which prevented either EGFL7 mRNA translation or caused the premature termination of translation. In both cases miR-126 could still be transcribed as a primary transcript containing the miR-126 is produced. Second and even more importantly, Parker et al. were able to rescue the phenotype they observed by the co-injection of EGFL7 mRNA together with a morpholino targeting EGFL7. This suggests that the tubulogenesis defect they observed was indeed caused by the loss of EGFL7 protein. Still, a potential interference of this knock-down strategy with the process of pre-miRNA formation and subsequent miR-126 maturation cannot be fully excluded at this stage.

Interestingly, a recent work demonstrated that EGFL7 is a direct target of the miR-126 in lung cancer cells and hinted that this could be at least a part of an explanation for the observed effect of miR-126 on tumourogenesis [8]. Likewise, Fish et al. described a transcriptional regulation of EGFL7 in human endothelial cells by miR-126 [6]. Taken together, above findings unambiguously demonstrate that miR-126 and EGFL7 share not only a structural but also a tight functional connection in different cellular contexts.

### Concluding remarks and future perspectives

Current data emphasizes a highly significant role of *egfl7 *for the development and homeostasis of the vascular system. The EGFL7 protein was shown to be an important factor governing lumen formation during vasculogenesis, whereas miR-126 harbored within the *egfl7 *gene played a crucial role in the process of angiogenesis and maintenance of vessel integrity. This way both molecules might very well collaborate to shape blood vessels.

Nevertheless, at the current stage there are many unresolved issues such as an imminent lack of a defined role of the EGFL7 protein in angiogenesis or an explanation of the mechanisms underlying EGFL7's effects on endothelial cells. Further, the functional relationship between the EGFL7 protein and miR-126 is not clear and one wonders if both act in synergy or antagonism. The undisputed role for EGFL7 in blood vessel formation beyond the function of miR-126 has yet to be proven and the underlying mechanism unraveled. Mice harboring a specific EGFL7 protein deletion without the confounding effect of the loss of miR-126 [51] do not show any abnormalities during angiogenic sprouting. This could potentially be explained by the upregulation of the EGFL7 homolog EGFL8 as indicated above. In this case it might prove useful to study EGFL7/EGFL8 double knock-out mice in order to understand the effects of the individual proteins on blood vessel formation. In addition, transgenic mice expressing EGFL7 under inducible conditions will help to shed light on the role of EGFL7 in angiogenesis.

Yet another interesting question to be addressed is the identification of novel EGFL7-interacting molecules on the surface of vascular cells. Given the fact that EGFL7 incorporates into the ECM, it seems possible that integrins mediate EGFL7's effects on endothelial cells, because integrins represent a class of receptors responsible for the interaction of virtually all types of adherent cells with the ECM. Integrins are indispensible for essential processes in the vascular system such as proliferation, migration or survival [61], plus, there is considerable data that suggest integrins to play a key role in the stimulation of tubular morphogenesis and the activation of endothelial cells [62,63]. This makes them prime candidates to be studied as mediators of EGFL7's effects on endothelial cells.

Further, it seems worthwhile to study the role of EGFL7 in cancer formation and progression as the role of *egfl7 *in angiogenesis points towards an interesting role of EGFL7 in tumor development. There have been several reports of EGFL7 expression in various tumors and tumor cell lines [4,5,64]. A recent study proposed EGFL7 increases the metastatic potential of human hepatocellular carcinoma by driving migration and invasion of tumor cells [64]. Taking into account the previously discussed data on the involvement of miR-126 in cancer it would be of interest to address the potential interplay of the EGFL7 protein and miR-126 within the tumor vasculature as well as the tumor cells themselves. Additionally, considering the dual effects of EGFL7 on endothelium and the tumor parenchyma, the development of novel agents targeting EGFL7 and its homolog EGFL8 may prove to be valuable additions to the already existing array of therapeutic strategies against cancer.

**Figure 5:**
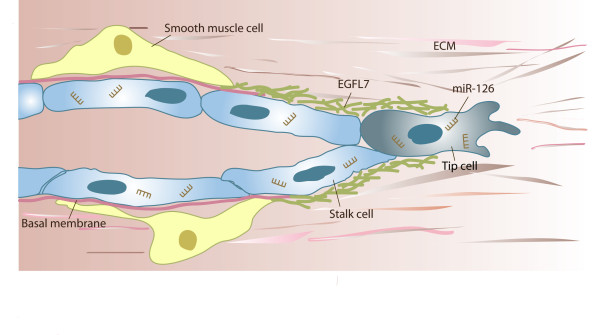
**Putative function of *egfl7 *in angiogenic sprouting**. Angiogenic sprouting proceeds through the coordinated actions of tip and stalk cells. Tip cells lead the sprout through the environment, while stalk cells proliferate and thereby contribute to sprout extension. Concurrently or subsequently to invasion, sprouts lumenize, assemble a basal membrane and eventually, recruit smooth muscle cells. EGFL7 is found incorporated into the provisional ECM surrounding the sprout, whereas miR-126 targets numerous mRNA molecules within the cell, which affects proliferation and the process of angiogenic sprouting.

In sum, *egfl7 *is a novel promising gene in vessel development. Current data emphasizes a role in vasculogenesis and angiogenesis by the modulation of endothelial and smooth muscle cells (Figure 5). Molecularly, these effects might be mediated by various signaling pathways. However, further work needs to address which of these possibilities apply and whether or not they all act in concert. As a modulator of vessel formation the *egfl7 *gene might prove useful for the treatment of vascular development disorders, cardiovascular diseases or cancer by tackling tumor neovascularization. Accordingly, the future is bright for the EGFL7/miR-126 alliance of angiogenesis.

## List of Abbreviations

Dll: delta-like; E: embryonic day; ECM: extracellular matrix; EGF: epidermal growth factor; EGFL7: epidermal growth factor-like domain 7 or epidermal growth factor-like domain, multiple 7; EGFL8: epidermal growth factor-like 8; LOX: lysyl oxidase; MEGF7: multiple epidermal growth factor-like domains protein 7; miR: microRNA; NICD: notch intracellular domain; SPRED-1: sprouty-related, EVH1 domain containing 1; PIK3R2: phosphoinositide-3-kinase, regulatory subunit 2; HUVEC: human umbilical vein endothelial cells; VE-statin: vascular endothelial statin

## Competing interests

The authors declare that they have no competing interests.

## Authors' contributions

IN and MS wrote the manuscript, KP made suggestions on the manuscript. All authors read and approved the final manuscript.
